# LncRNA & Wnt signaling in colorectal cancer

**DOI:** 10.1186/s12935-020-01412-7

**Published:** 2020-07-20

**Authors:** Zeeshan Javed, Khushbukhat Khan, Haleema Sadia, Shahid Raza, Bahare Salehi, Javad Sharifi-Rad, William C. Cho

**Affiliations:** 1 Office for Research Innovation and Commercialization, Lahore Garrison University, Sector-C, Phase VI, DHA, Lahore, Pakistan; 2 Atta-ur-Rahman School of Applied Biosciences (ASAB), National University of Sciences and Technology (NUST), Islamabad, 44000 Pakistan; 3 Department of Biotechnology, Balochistan University of Information Technology, Engineering and Management Sciences, Quetta, Pakistan; 4 Department of Biotechnology, University of Central Punjab, Lahore, Pakistan; 5 Noncommunicable Diseases Research Center, Bam University of Medical Sciences, Bam, Iran; 6 Student Research Committee, School of Medicine, Bam University of Medical Sciences, Bam, Iran; 7 Phytochemistry Research Center, Shahid Beheshti University of Medical Sciences, Tehran, Iran; 8 Department of Clinical Oncology, Queen Elizabeth Hospital, 30 Gascoigne Road, Hong Kong, China

**Keywords:** *Wnt* signaling, Colorectal cancer, lncRNA, Therapeutics strategy

## Abstract

The outlook for new therapeutic approaches is pivotal to ameliorate the deterioration caused by the abrogated *Wnt* signaling. Long non-coding RNAs (*lncRNAs*) are tiny molecules that have begun emerging as vital molecular manager for the regulation of various cellular processes at transcription and translation levels in the colorectal cancer (CRC). Targeting *Wnt* pathway with lncRNA seems a promising approach to eradicate CRC. However, little is known of their active role in commencing both apoptosis and proliferation in CRC. This article  reviews the importance of these molecules in the pathogenesis of CRC and also emphasizes on the development of new therapeutic strategies to cope with the *Wnt* mediated CRC.

## Background

Colorectal cancer (CRC) is the third most prevalent maligancy in the world. It has been estimated that there are 1.8 million cases of CRC reported globally and the figure will project to have an upsurge to 2.6 million in the next decade [1]. CRC incidence ranges from 6.5 per 100,000 in the middle east and Africa to 83.7 per 100,000 in Asia pacific. However, the incidence of CRC is highest among Asian countries with 737,000 cases reported each year [2]. The new therapeutic interventions is pivotal for disease management. Despite the advancement in the field of precision targeting of cancer with small molecules, the treatment of CRC is still bleak. Unavailability of validated molecular and phenotypic targets has greatly stalled the efficacious treatment of CRC. Monoclonal antibodies targeted against epidermal growth factor receptor (*EFGR*) and vascular endothelial growth factor (*VEGF*) have gleaned some success. However, poor prognosis in advanced CRC has greatly hampered their effective use [3]. High-throughput technologies, e.g. next-generation sequencing have begun to scratch the surface of the mutations that drive intestinal epithelial cell transformation and carcinogenesis in the CRC. Mutations associated with de-regulated *Wnt*-signaling cascade entails severe proliferative characteristics in various subtypes of CRC and thus are the promoting factors in many forms of CRC [4]. So far, mutations of adenomatous polyposis coli (*APC*) have been enlisted as the primal genetic event that leads to tumorigenesis in the CRC [5]. Also, a series of mutation drivers that deregulate the machinery are involved in regulating cell growth, development and differentiation of CRC [6]. Oncogenic mutations in the *Wnt* pathway are a hallmark in CRC. More than 80% CRC tumors carry inactivating mutations in the regulatory component of *Wnt* pathway such as the *APC*. *APC* have been implemented to be a decisive factor in the malignant transformation of the CRC epithelial cells [7]. Long non-coding RNAs (lncRNA) are small molecules that have been implemented to orchestrate plethora of cellular processes. Involvement of *lncRNAs* in modulation of gene expression and regulation of signaling cascades has been a spotlight over the years [8]. LncRNAs are small molecules by size > 200 bp. Advances in the field of molecular biology have begun to unravel the mysteries of *lncRNAs* in various cancers. *LncRNAs* possess unique properties that separate them from the coding RNAs [9]. The majority of CRCs is driven by the oncogenic mutations in *Wnt* pathway [10]. Despite the influential role of *Wnt* mutations in the CRC, the role of *lncRNAs* in regulating the *Wnt* signaling cascade in CRC is still dreary [3]. Exploring *lncRNAs* as a therapeutic target for *Wnt* mediated abrasions in CRCs is a promising strategy. Here we give a brief review of *lncRNAs* involved in *Wnt* pathway that may be targeted in the CRC.

### Wnt signaling pathway in CRC

*Wnt* pathway has been reported to orchestrate differentiation and development of metazoan via modulation of the key transcriptional framework [6]. Both canonical and non-canonical signaling of the *Wnt* Pathway contributes to the development and differentiation [11]. In case of canonical *Wnt* signaling (also referred to as *Wnt*/*β*-*catenin* signaling), the downstream processing is controlled by a squad of context-specific afferent ligands such as the frizzled (*fzd*), phosphor tail of *LRP5/6* and disheveled protein (*Dsh*). *Wnt*/*β*-*catenin* signaling cascade expression is strictly guided by *β*-*catenin* destruction complex consisting of *APC* (adenomatous polyposis coli), *Axin2*, casein kinase (*CK1*) and glycogen synthetase kinase β (*GSK β*) [12, 13]. *β*-*catenin* destruction complex explicitly monitor degradation of *β*-*catenin* through proteasome-mediated destruction complex, comprising of protein E3 ubiquitin ligase (beta-transducin repeat containing E3 ubiquitin protein ligase) *β*-*TRCP* [14]. The destruction complex phosphorylates the *β*-*catenin* at serine/threonine residues at the N-terminus which facilitates ubiquitination by *β*-*TRCP* for proteasome recruitment [13]. Presence or absence of *APC* is a defining factor in the working of the destruction of the complex. *APC* safeguards covalent modifications of *β*-*catenin* that ensure scaffolding and the assembly of the destruction complex [15]. However, the activation of *Wnt* signaling cascades by sequential ligands (*Dsh, LRP5/6* and *Fzd*) efficiently hampers ubiquitination by the destruction complex. Furthermore, these context specific ligands promote *β*-*catenin* to stabilize and interact with various transcription factors such as the (Tcell factor and lymphoid enhancer factor) *TCF/LEF* (T cell factor/lymphoid enhancer factor) family of transcription factors. Recruitment of transcription factors of target genes greatly enhances the *Wnt* pathway target gene expression [16].

### LncRNAs: the mediators and modulators of cancer

The human genome is more intrusive as a vast portion of the genome is not transcribed into proteins. This non-coding genome was once referred to as junk DNA [17]. However, genome wide analysis approaches and high throughput technologies have begun to delineate the mysteries of the non-coding genome. *LncRNAs* which are comparatively larger than the microRNAs have started to emerge as a potent player in the cancer biology [18]. Non-coding RNAs share certain homologies with the coding genome, yet they have their own significant features that part them from the coding genome. Based on genomic peculiarities non-coding RNAs are divided into long non-coding RNAs (*lncRNAs*), long intergenic non-coding RNA (*lincRNA*), ultra-conserved regions (*T*-*UCRs*), enhancer RNA (*eRNAs*), circular RNA (*c*-*RNAs*), Promoter associated RNAs and several others [19]. Based on their functions *lncRNAs* can act as (i) signal transducer; (ii) molecular decoy; (iii) molecular Sponge; (iv) Cis–Trans activator and; (v) chromatin remodeler [20].

Plethora of studies has shed light on the crucial role of lncRNAs in CRC. Wnt signaling is a crucial mediator of cellular growth, proliferation, invasion and metastasis. The interaction between Wnt-signaling and lncRNAs seems to be promising approach to understand the complex nature of CRC, Here we explain the interplay between lncRNAs and Wnt-signaling on the basis of different functions (signal transducer, molecular decoy, molecular Sponge, Cis–Trans activator, and chromatin remodeler). We have attempted to explain how lncRNAs interplay with Wnt-signaling to trigger growth or apoptosis and how can lncRNAs be used as prognostic or diagnostic marker for early and rapid detection of CRC.

### Interplay between lncRNAs and Wnt-Signaling in CRC

**Fig. 1:**
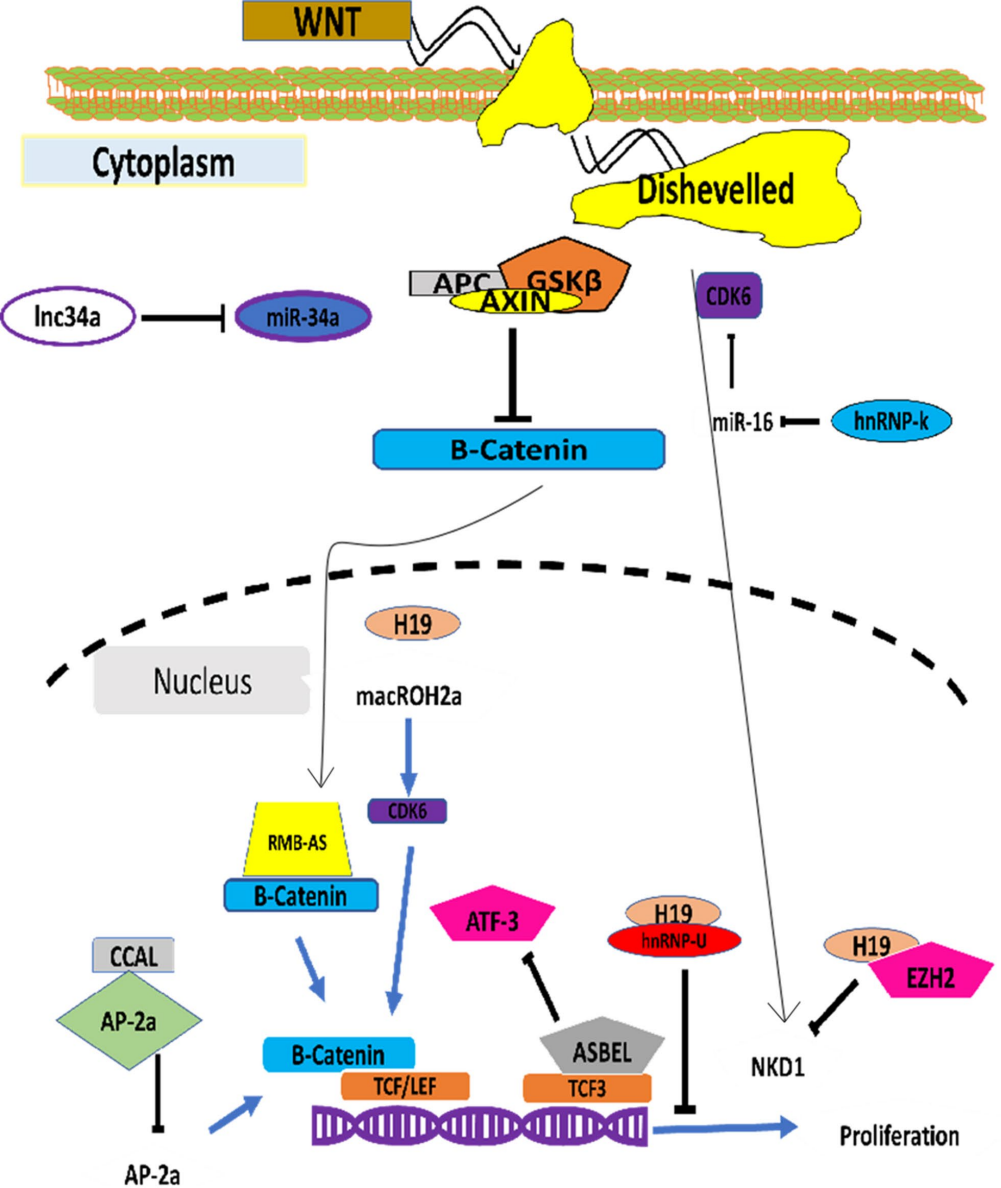
Interplay between *Wnt* signaling cascade and *lncRNAs*: in canonical *Wnt* signaling several *lncRNAs* interact with *Wnt* consequently affect the expression of the targeted genes. Arrows indicate activation and inhibition by the *lncRNAs*

Molecules involved in *Wnt* signaling directly or indirectly affect gene expression by acting as a transcription factor or modulating the expression of other transcription factors. Furthermore, several studies have demonstrated the involvement of *lncRNAs* in the regulation of the *Wnt* signaling cascade at both transcriptional and translational levels [21]. Wnt signaling is indispensable for a plethora of cellular processes which includes tumor proliferation, metastasis and stemness [22]. Wnt signaling orchestrates these cellular processes in CRC. In addition, Wnt signaling cascade plays a pivotal role in maturation, differentiation and development of the both normal and cancer stem cells [22, 23]. LncRNA have begun to scratch the surface of the essential regulatory machinery involved CRC [24]. The interplay between lncRNA and Wnt signaling cascade is brimming with opportunities to delineate the role of these micro managers in development, differentiation and metastasis of CRC (Table 1). LncRNAs possess many advantages as majority of lncRNAs associated with Wnt signaling in CRC shed light on prognosis [25]. Therefore, they may be used as powerful diagnostic approach for the early detection of the CRC. The modulation of wnt signaling cascade through lncRNAs is illustrated in Fig. 1. Recent study has demonstrated the role of PCA3 a lncRNA in prostate cancer. Expression analysis in conjunction with disease progression indicated that lncRNA could be implemented as tissue specific cancer biomarker for prostate cancer [26]. In addition to this expression of a specific lncRNA is tissue specific and often is under the influence of specific genes, consequently lncRNA can be explored as biomarker as it has been exemplified using the CCAT-1 expression in response to BRD4 in clinical trials. Since many lncRNAs explored so far have genetic predisposition towards tumor. Their interaction with DNA or RNA can predict the out-come of disease and thus can be implemented as biomarker for SNPs mediated tumor anomalies [27]. LncRNAs regulate Wnt-signaling cascade thus, they can evince to be valuable therapeutic option for the treatment of various cancers. In addition to this role of PVT1 in fine tuning the expression of the c-MYC protein can be utilized as source for therapeutic interventions [28]. Lnc34a is another lncRNA that can be perceived as a therapeutic tool against CRC as it has been involved in the regulation of CRC stem cells. Furthermore, lnc34a ameliorates the aggravated cancer stem cells growth. Therefore, lnc34a role in CRC physiology must not be neglected and should be explored for devising therapeutic strategies for CRC. Despite their prolific role in development and differentiation of cells, there are certain limitations that have still impeded progress related to lncRNA as therapeutic strategy for various cancers [29]. However, advancements in the field of high-throughput technologies, and genome wide sequencing has begun to narrow the gaps related to lncRNA biology. It has now become plausible to circumscribe the expression pattern and tissue related expression of lncRNA. In addition to this development in the field of siRNA, anti-sense oligonucleotides (ASO) have made it easy to study the functionality of the lncRNA in different organs and tissues. lncRNA mediated silencing of target genes is an unmet challenge in CRC, therefore development of target specific ASO with limited side effects is worth exploring.

**Table 1:** LncRNAs involved in Wnt signaling in CRC

LncRNA	Targeted Wnt pathway	Effects
CCAT1-S CARLo-5	TCF4 expression through up-regulation of the SNPrs6983267	Poor prognosis and low survival rate in CRCs
CCAT-1-L	Interacts with the chromatin modeler CTCF and MYC expression	Growth and differentiation of CRC
CCAT-2	Regulates the TCF7L2 expression through up-regulation of the SNPs	Poor prognosis and low survival rate in CRCs and promotes metastasis and proliferation
CASC11	Activates the expression of Beta-Catenin and modulates the expression of MYC gene through SNP rs16902359	Elevates tumorigenesis and metastases of primary tumors to lymph-nodes in CRC
PVT1	Acts as scaffold RNA site that covers Threonine residue 58 and controls a range of miRNAs such as the miR-1204, miR-1205, miR-1206, miR-1207-5p, miR-1207-3p and miR-1208	Poor prognosis and low survival rate in CRCs and promotes metastasis and proliferation
Lnc34a	Regulates the expression of miRNA-34a which acts on TCF7L2 and beta-catenin and Notch signaling	Asymmetric growth of CRC stem cell
RBM5-AS1	Beta-Catenin and SGK1, YAP1 and MYC via TCF7L2 transcription factor	Growth and proliferation of CRC stem cells in vivo and in vitro
ASCL2	Beta-catenin production and interaction with TCF7L2	Growth and stemness of CRC stem cells and intestinal cells
WiNTRLINC1	Beta-Catenin and ASCL2 expression	Poor prognosis and low survival rate in CRCs and promotes metastasis
PCAT1	Regulates the expression of LGR5 gene and Wnt signaling to promote the expression of MYC	Poor prognosis and low survival rate in CRCs and promotes metastasis
lncRNA H19	Regulates the expression of MYC and beta-catenin through CDK8 production	Growth and proliferation of CRC
CCAL	β-catenin/TCF7L2 and multidrug resistance gene 1 (MDR-1) and modulates the expression of AP2α	Poor survival, devastating metastasis and resistance to adjuvant chemotherapy in CRC

### Human 8q24 “Gene desert” the hotspot for CRC

The human 8q24 gene desert is a hub for many *lncRNAs* which have been implicated to monitor *Wnt* signaling cascade [30]. Expression analysis studies have confirmed the presence of colon cancer associated Transcript 1 (*CCAT*-*1*), *CCAT*-*1*-*L*, *CCAT*-*1*-*S*, *CCAT*-*2*, *CASC11* and several other *lncRNAs* in this region [31]. The details of this region in regulating the growth and differentiation have been discussed here:

**Colon cancer associated Transcript 1 (*CCAT*-*1*)** also known as *CARLo*-*5* is a gene located in close vicinity of the *MYC* oncogene [31]. There are two variants, namely *CCAT*-*1*-*S* and *CCAT*-*1*-*L,* encoded by the *CCAT*-*1* gene. *CCAT*-*1* has been reported to be upregulated in almost all stages of CRCs and its overexpression usually curtails poor prognosis and low survival rate in CRCs patients [31]. *CCAT*-*1* overexpression and tumor progression has also been linked to other tumors such as breast cancer [32]. The isoform *CCAT*-*1*-*S* has been reported to regulate *Wnt* signaling effector *TCF4* expression through up-regulation of the SNPrs6983267 [28, 33]. *rs6983267* is in the telomeric region of the *CCAT*-*1*-*S* isoform which is activated through the loop formed by the *CCAT*-*1*-*S*. Knock-down of *CCAT*-*1*-*S* significantly hampered CRC growth both in vivo and in vitro [32]. Furthermore, *CCAT*-*1*-*L* has also been implemented to enhance cellular growth and proliferation of the CRC. *CCAT*-*1*-*L* overexpression has been found in several CRCs and xenograft mouse models [34]. *CCAT*-*1*-*L* interacts with the *MYC* gene at its promoter and facilitates effective transcription. *CCAT*-*1*-L interacts with the chromatin modeler *CTCF*, interaction between *CTCF* and *CCAT*-*1*-*L* trigger chromosomal unwinding that in turn promotes the effective transcription of *MYC* and thus growth and differentiation of CRC [34]. From these findings, it can be deciphered that *CCAT*-*1* acts as enhancer RNA that promotes the active transcription of *MYC* [34, 35]. This molecular interaction is complex as it involves the SNP, enhancer RNAs and modulation of *MYC* expression that ultimately leads to growth of CRC [35].

***CCAT*-*2*** is another lncRNA that has been investigated for its role in the progression and metastasis of CRC [36]. *CCAT*-*2* has been implemented to play a decisive role in the progression of tumor by several ways. Genomic region encompassing *CCAT*-*2* is highly conserved throughout the species, makings it indispensable for the transcription [37]. This region also contains the SNPrs6983267 a highly predisposing single nucleotide polymorphism affiliated with the CRC and prostate cancer [38]. Furthermore, *CCAT*-*2* lies very close to the *MYC* gene, thus, *CCAT*-*2* has a role in the Cis regulation of *MYC* gene [33]. *CCAT*-*2* upregulation has been reported to enhance tumor progression and metastasis in microsatellite stable CRCs [39]. Additionally, these tumors also exhibited high levels of chromosomal instability. However, *CCAT*-*2* was found downregulated in the microsatellite instable tumors with limited levels of Chromosomal instability [19]. These findings suggested that *CCAT*-*2* expression was pivotal for enhancing the CRC progression in vitro [39]. Accumulating data have begun to suggest the involvement of *CCAT*-*2* in trans regulation of the *Wnt* signaling cascade. The transcription factor like 7 L2 (*TCF7L2*) a transcription factor of *Wnt* signaling cascade is activated by the *CCAT*-*2* lncRNA in the presence of DNA elements and enhancers and also under the influence of the SNPs [39]. The absence of DNA elements, scarcity of the enhancer and berated expression of SNPs greatly hampered the growth of intestinal cells of CRCs in vivo [39]. Recently, this non-coding RNA was reported to exert allele-specific effects on cancer metabolism by interaction with the splicing protein CFIm and ensuring alternative splicing of glutaminase [40]. In addition, multiple meta-analysis studies reported the prognostic value of *CCAT*-*2* in predicting cancer patient survival [39].

***Carlo*-*7*** also known as the *CASC 11* is another lncRNA that has been investigated recently for its involvement in tumor progression. *CASC11* is also a neighboring gene of the *MYC* [41]. This region also contains a single nucleotide polymorphism SNP rs16902359. *CASC11* up-regulation is a hallmark in CRC tumor biology [41]. Overexpression of CASC1 curtails elevated tumorigenesis and metastases of primary tumors to lymph-nodes. Furthermore, *CASC11* overexpression promotes the stability of heterogeneous ribonucleoprotein K (*hnRNP*-*K*) [41]. Stable *hnRNP*-*K* prevents the proteasome mediated destruction of *β*-*catenin* in a feedback response manner and consequently, promote growth and differentiation of the tumor cell. *CASC11* expression is modulated by the *MYC* protein. *MYC* protein binds to the promoter region of the *CASC11* that facilitates *CASC11* expression [41].

***PVT1*** also known as *pvt 1* oncogene is located downstream of the *MYC* gene and regulate translation of *MYC* gene [42]. *PVT1* inhibits degradation of *MYC* gene by preventing phosphorylation at serine threonine residues. New studies have begun to shed light on the interaction between *MYC* protein and *PVT1* RNA [42, 43]. *PVT1* lncRNA acts as scaffold RNA site that covers Threonine residue 58 [42]. Thus, prevent phosphorylation and promote growth and differentiation of the CRC cells. *PVT1* act as oncogene that facilitates the expression of *MYC* is confirmed by chromosome engineering. Both *MYC* and *PVT1* are expressed in equal amounts in the HCT116 cell lines. The reduction of *PVT1* significantly reduced the cell growth in HCT116 cell lines [42]. while *PVT1* overexpression has been linked to poor prognosis in patients with CRC [43]. Therefore, it can be used a diagnostic marker for the CRCs. The *PVT1* gene cluster houses large number of miRNAs such as, *miR*-*1204*, *miR*-*1205*, *miR*-*1206*, *miR*-*1207*-*5p*, *miR*-*1207*-*3p* and *miR*-*1208* [44]. Their functionality is still bleak however; exploring the role of these miRNAs will enhance our understanding of *PVT1* mediated tumor progression.

### LncRNAs and Wnt signaling in CRC stem cell development

*Wnt* mediated differentiation of the cancer stem cells confer resistance to several drugs in CRC [45]. Furthermore, targeting this derailed *Wnt* signaling with *lncRNAs* is a promising new strategy to be explored. Several *lncRNAs* have been reported that efficiently orchestrate the CRC stem cells development and differentiation. Advancements in the field of genome wide analysis and high throughput technology have begun to reveal the depth of tumor biology.

**Lnc34a** has been implicated to suppress the expression of the miR-34a as it is transcribed in anti-sense orientation. Like  other cancers, CRC has been reported to contain loss of function mutation of *miR-34a* [46]. *miR*-*34a* has been involved in regulation of *Wnt* signaling cascades at both transcriptional and translational levels [47]. *miR*-*34a* directly targets the *β*-*catenin* and promotes its proteasome mediated degradation [47]. *miR*-*34a* also reduces the expression of *Wnt* targeted gene by interacting with the transcription factor *TCF7L2* [48]. These interactions hamper the *Wnt* mediated differentiation of the tumor cells [49]. It has come to light less lately that *miR*-*34a* also interacts with the Notch signaling pathway and directly triggers the asymmetric growth of CRC stem cell via a feedforward loop with Numb proteins [50]. New finding has revealed the involvement of a lncRNA (*lnc34a*) in modulation of the expression of the *miR*-*34a*. Furthermore, *lnc34a* interactions with epigenetic regulators DNA methyltransferase 3a (*Dnmt3a*), histone deacetylase 1 (*HDAC1*) and *PHB2* successfully hinder the expression of the *miR*-*34a* in the absence of *P53* protein. Altogether, these findings indicate the important role of *lnc34a* in modulation of *miR*-*34a* expression and in growth and differentiation of the CRC stem cells [51]. However, *lnc34a* has a dual role in development of CRC stem cells. In mice model, it has been delineated that *lnc34a* promotes the growth of CRC stem cells via inhibition of *miR*-*34a* expression but the growth was limited to only first daughter cells [51]. Moreover, *lnc34a* was found to be unequally divided in the CRC stem cells and this unequal distribution promoted the suppression of *miR*-*34a* in one daughter cells while increase in the growth of the other CRC stem cells [51]. *Lnc34a* has also been found to upregulate the in the later stages of CRC growth indicating the fact that miRNA-lnRNA interplay is crucial for the development and differentiation of CRC [51]. The functional importance of the lncRNA-miRNA interaction can glean the structural framework that potentiates growth promoting pathways such as the *Notch* and *Wnt*.

LncRNA known as the ***RBM5*-*AS1*** is reported to be up-regulated during the early stages of the CRC [52]. ASOs and siRNA-based approaches applied to silence this lncRNA resulted in down-regulation of the *Wnt* Signaling cascade and in turn minimal growth of CRC [52]. However, forced expression of *RBM5*-*AS1* promptly increased the cell division in early stage CRC Stem cells. Functional analysis studies confirmed that *RBM5*-*AS1* directly interacts with the *β*-*catenin* and thus, facilitate the expression of oncogenes (*SGK1*, *YAP1* and *MYC*) under the influence of transcription factor *TCF7L2* complex [52].

*Wnt* signaling plays a crucial role in maintaining the stemness of intestinal cells by interacting with transcription factors such as the ***ASCL2*** [53]. *β*-*catenin* is released from receptor ligand interaction progressively migrates to the nucleus where it interacts with the transcription factor TCF7L2 in the presence of *ASCL2* [54]. This in turn promotes consistent cell growth of intestinal cells. Chip-sequencing data has demonstrated the role of a lncRNA in promotion of the cancer stemness in the intestinal cells by modulating the expression of *ASCL2* [54]. Newly defined lncRNA ***WiNTRLINC1*** (*Wnt*-regulated lincRNA-1) has been reported to interfere with the expression of the *ASCL2*. Chip-seq data confirmed that *WiNTRLINC1* facilitates the recruitment of *β*-*catenin*/TCF7L2 regulatory elements close to the *ASCL2* via formation of loop that comprehensively aids in maintenance of stemness in the intestinal cells. Expression analysis studies have confirmed overexpression of *WiNTRLINC1*in CRC tumors [54]. The interaction between *β*-*catenin* and *WiNTRLINC1* resulted in increased invasiveness, metastases and poor prognosis in the CRC.

***lncTCF*** is another lncRNA that plays a pivotal role in the development and differentiation of the cancer stem cells [24]. However, its role as oncogenic lncRNA in CRC has less come to light, lncTCF endorse the expression of the Wnt signaling cascade that in turn promoted the growth of the CRC cells [24]. Increased expression of the lncTCF has been affiliated with the poor prognosis and increased differentiation of the CRC [24].

Another lncRNA ***PCAT1*** has also been enlisted to enhance proliferation of the CRC via up-regulation of the MYC [45]. PCAT1 overexpression leads to poor prognosis and low survival rates in patients with CRC [46]. Wnt signaling cascade is indispensable for tailoring the new outlook for both the normal as well as cancer stem cells [55, 56]. lncRNAs curtails the oncogenic effects of Wnt signaling by regulating the expression key proteins at both transcription and translation levels [57]. It has been well established that aberrant Wnt signaling mediated regulation of LGR5 gene trigger stem cell differentiation and asymmetric cell division.

### LncRNAs as activator of proliferation, metastasis and invasion in CRC

**lncRNA** ***H19*** has been reported to inflict death punches in CRC. *H19* was discovered in early 90 s and its presence is instrumental in embryonic development [58]. *H19* involvement in various cancers has been well documented [59]. *H19* interplays with the variety of miRNAs either inhibiting their expression (*let7* and *miR*-*106a*) or facilitating their transcription (miR-675) [60]. A recent study has demonstrated that enhanced expression of ***H19*** **lncRNA** was related to poor prognosis of various human cancers. Suppression of *H19* resulted in increased cell survival and reduced migration in CRC [61]. *H19* interaction with the miRNA let-7 and *MYC* is well known for tumor progression, however, recently microarray-based study has surfaced the interplay between *H19* and *β*-*catenin*, that triggers growth in Hepatocellular carcinoma via up-regulation of the *CDK8* expression [62, 63]. Further, *H19* acts as catalyst that superficially regulates the expression of *Wnt* targeted genes as well as the *MYC* gene in many cancers [61–63]. Altogether, these findings suggest *H19* as an exclusive interpreter in stimulating as well as inhibiting the cellular growth. *H19* interaction triggers the *CDK8* production through Histone modifications. It interacts with the hnRNP and prevents the downstream signaling of *Wnt* pathway that leads to differentiation suppression of the liver cells [64]. From these perspectives it is clearly demonstrated that *H19* lncRNA although very small in size, can prove out to be possible diagnostic marker for CRCs. HI9 has been reported to effect miRNA let-7 activity, induce regulation of CDK8 through interplay with β-catenin and monitor the methylation at genome wide levels [65]. These findings suggested that the lncRNA H19 as therapeutic candidate for targeting Wnt mediated signaling cascade in CRC.

**lncRNA** ***CCAL*** intensify the overall CRC progression. Poor survival, devastating metastasis and resistance to adjuvant chemotherapy are the salient features associated with *CCAL* [66]. *CCAL* confer chemotherapy resistance by indirectly modulating the expression of *Wnt* targeted genes such as *β*-*catenin*/*TCF7L2* and multidrug resistance gene 1 (*MDR*-*1*). *CCAL* interacts with *AP2α* protein and promotes the degradation of *AP2α,* a negative regulator of *β*-*catenin*/TCF7L2 interaction, in CRC, and thus indirectly activates *Wnt* signaling [66]. This in turn promotes the expression of MDR1. MDR1 encodes the protein P-glycoprotein 1 whose overexpression facilitates drug resistance in majority of CRC [66].

***CTD903*** is another lncRNA whose ectopic expression has been related to mitigate growth of the CRC [67]. However, its downregulation resulted in the up-regulation of the *β*-*catenin* which consequently, promoted growth and differentiation of CRC in vivo [67]. Furthermore, *CTD903* has been investigated for its role in the promotion of the epithelial-mesenchymal transition (EMT). *CTD903* reduced expression promoted EMT in CRC with an increased expression of transcription factors TWIST, SNAIL and vimentin. Additionally, there was reduction in the expression of epithelial marker ZO-1 [67]. However, the exact mechanism that elaborate the *CTD903* downregulation promote *Wnt* mediated EMT remains to be explored.

***BTG3*-*AS1*** also referred to as *ASBEL* is another lncRNA that has been implemented to play crucial role in CRC proliferation and metastasis [68]. A recent study using Chip-Seq and RNA-seq confirmed that the knock down of the *ASBEL* greatly reduced tumor growth in mice model. *ASBEL* interacts with the Transcription factor 3. *TCF3* is direct target of *β*-*catenin*. *β*-*catenin* interaction with the *TCF3* promotes the transcription of the *ATF3* that accentuate tumor proliferation [68]. It can be concluded that *ASBEL* interaction with *Wnt* signaling cascade promote tumor progression in CRC.

***lncRNA GAS5*** (growth arrest specific 5) has been investigated to play a crucial role in prevention of angiogenesis, invasion and metastasis of the CRC [69]. A recent study shed light on the anti-proliferative aspects of *GAS5* in CRC. *Wnt* signaling abrasion is a hall mark of CRC and *GAS5* overexpression hampers the angiogenesis of the CRC [69]. Small interfering RNA approach confirmed that the inhibition of the *GAS5* resulted in elevated growth and angiogenesis in CRC cell lines [69]. However, the exact mechanism by which *GAS5* inhibits *Wnt* signaling cascade is still austere.

A recently characterized lncRNA, ***lncRNA*-*APC1*** has been evidenced to play a decisive role in the pathogenesis of the CRC [70]. Microarray based study confirmed that down regulation of *lncRNA*-*APC1* was significant to exacerbate metastasis and invasiveness of CRC [70]. However, forced expression of *lncRNA*-*APC1* significantly hampered cellular growth and differentiation of the CRC. Furthermore, *APC1* expression was sufficient to inhibit CRC cell growth, metastasis, and tumor angiogenesis by suppressing exosome production through the direct binding of Rab5b mRNA and a reduction of its stability. Importantly, exosomes derived from *lncRNA*-*APC1*-silenced CRC cells promoted angiogenesis by activating the *MAPK* pathway in endothelial cells [70]. These findings shed lights on the implementation of the *lncRNAs* for a diagnosis as well as therapeutics in CRC.

**LncRNA** ***SLCO4A1*-*AS1*** has been reported to relate to tumor proliferation and metastasis [71]. Knock-down of *SLCO4A1*-*AS1* resulted in overall greater proliferation and metastasis in vitro. *SLCO4A1*-*AS1* directly interacts with the *β*-*catenin* and facilitates the expression of oncogenes. The Glycogen synthetase kinase β (*GSKβ*) inhibits the expression of *β*-*catenin* and also inhibitsproliferation [71]. However, *SLCO4A1*-*AS1* interacts with *β*-*catenin*, stabilizes *β*-*catenin* and prevents its phosphorylation by the *GSKβ* that in turn promote differentiation, invasion and metastasis of CRC cells [71].

***NEAT1*** has also been reported to play a role in CRC progression. A recent study has delineated the mechanism responsible for the CRC under the influence of *NEAT1* [72]. *NEAT1* indirectly promotes the activation of *Wnt* signaling cascade through activation of the Death domain Protein (*DDX5*). Upregulated *NEAT1* interaction with the *DDX5* protein triggers the *Wnt* signaling in CRC. This indirect activation promotes metastasis and invasiveness of CRC [72]. This study signifies the importance of *NEAT1/DDX5/Wnt* cascade as therapeutic target for the treatment of the CRC.

**lncRNA zinc finger E-box binding homeobox 2 anti-sense 1 (*ZEB1*-*AS1*)** has been reported to act as oncogenic lncRNA that promote cell proliferation [73]. *ZEB*-*AS1* knockdown resulted in lower rates of proliferation and increased apoptosis. Furthermore, bioinformatic data suggest that *miR*-*181a*-*5p* has a role in orchestrating the function of *ZEB*-*AS1*. *miR*-*181a*-*5p* negatively modulated the expression of the *ZEB*-*AS1* as confirmed by the luciferase and RIP assay. This study suggested that *ZEB*-*AS1*, act as a molecular sponge for *miR*-*181a*-*5p* [73]. These findings revealed that ZEB-AS1 is an oncogenic lncRNA that promotes proliferation of CRC cells.

qRT-PCR and knock down expression analysis revealed the involvement of HOX transcript antisense RNA (***HOTAIR***) in proliferation and chemoresistance of CRC [22]. *HOTAIR* is generally up-regulated in the CRC tumors as compared to normal tissue and its overexpression deterred apoptosis. However, *miR*-*203a*-*3p* overexpression elevated apoptosis and inhibited proliferation in vitro [74]. *HOTAIR* promoted the expression of the *β*-*catenin* and *TLE* family related transcript protein *GRG5* via inhibition of *miR*-*203a*-*3p*. *HOTAIR* mediated overexpression of the *β*-*catenin* triggered cell growth and chemoresistance. The overexpression of *miR*-*203a*-*3p* significantly reduced the proliferation and chemoresistance [74]. These findings suggested the oncogenic role of *HOTAIR* in the development of the CRC.

Small nuclear host gene 1 (***SNHG1***) is another lncRNA whose upregulation enables CRC progression and metastasis [75]. Recent in vitro experiments have indicated that up-regulation of the *SNHG1* promotes the expression of the *β*-*catenin* and transcription factor-4 (*TCF*-*4*). In addition to this *SNGH1* has also been implemented to elevate the expression of the *Cyclin D1* and *membrane metalloprotease*-*9* [25]. These findings indicated that *SNGH1* is upregulated in CRC and act as an oncogenic lncRNA which can be used as a diagnostic marker as well as a therapeutic target for CRC.

**lncRNA** ***linc00675*** has been reported for inhibiting proliferation and metastases in different cancers. However, a recently surfaced study has delineated *linc00675* role in CRC. This finding suggested that *linc00675* has an anti-proliferative role in CRC. *linc00675* interplays with the *miR*-*942* and prevents proliferation and metastases. *linc00675* was found to be expressed in all CRC tissues and prevented the expression of *miR*-*942*, however, *miR*-*942* mimics significantly increased the expression of *Wnt*/*β*-*catenin* pathways and increased differentiation in vivo [76]. These studies shown the importance of *linc00675* as an potential diagnostic and therapeutic target for CRC.

## Conclusion

*Wnt* signaling controls a plethora of cellular processes in CRC. Cancer cell growth, differentiation, metastasis, drug resistance and disease relapse are tightly influenced by the abraded *Wnt* signaling. Therefore, *Wnt* signaling cascade is indispensable for the tumor cell maintenance. lncRNAs are tiny molecules that interact with the *Wnt* signaling in various ways. Interaction between lncRNA, *Wnt* signaling and corresponding molecules results in the orchestration of various cellular processes responsible for cell fate determination, development, differentiation and metastasis. The exact mechanism responsible for governing these interactions between lncRNA and *Wnt* is still bleak and requires more industrious efforts to delineate these mechanisms. This has become a stumbling block in the voyage for discovering new therapeutics for CRC. Recent advances in microarray, single molecule visualization techniques, RNA-sequencing, and methylation profiling will enable us to identify the hidden mysteries of lncRNA biology and interaction with *Wnt* signaling in CRC. Furthermore, clinical significance of *lncRNAs* in CRC can be illustrated by the use of large-scale database analysis. More efforts are required to understand the primary, secondary and tertiary structures of the lncRNAs as it will enable us to forecast structural and functional interactions at molecular levels. Furthermore, exploring the interplay between lncRNA and *Wnt* will bring us a step closer towards new therapeutic breakthroughs in CRC. Also, the advances on mechanism of understanding *lncRNAs* in *Wnt* signaling might bring novel candidates as biomarkers and therapeutics for CRC. Furthermore, single-cell sequencing may enable us to design oligonucleotide-based drugs and will help to achieve the potential of lncRNA as an alternative cancer therapy.

## Data Availability

Yes.
